# Nucleotide excision repair of aflatoxin-induced DNA damage within the 3D human genome organization

**DOI:** 10.1093/nar/gkae755

**Published:** 2024-09-11

**Authors:** Yiran Wu, Muhammad Muzammal Adeel, Dian Xia, Aziz Sancar, Wentao Li

**Affiliations:** Department of Environmental Health Science, College of Public Health, University of Georgia, Athens, GA 30602, USA; Department of Environmental Health Science, College of Public Health, University of Georgia, Athens, GA 30602, USA; Department of Environmental Health Science, College of Public Health, University of Georgia, Athens, GA 30602, USA; Department of Biochemistry and Biophysics, University of North Carolina School of Medicine, Chapel Hill, NC 27599, USA; Department of Environmental Health Science, College of Public Health, University of Georgia, Athens, GA 30602, USA

## Abstract

Aflatoxin B1 (AFB1), a potent mycotoxin, is one of the environmental risk factors that cause liver cancer. In the liver, the bioactivated AFB1 intercalates into the DNA double helix to form a bulky DNA adduct which will lead to mutation if left unrepaired. Here, we adapted the tXR-seq method to measure the nucleotide excision repair of AFB1-induced DNA adducts at single-nucleotide resolution on a genome-wide scale, and compared it with repair data obtained from conventional UV-damage XR-seq. Our results showed that transcription-coupled repair plays a major role in the damage removal process. We further analyzed the distribution of nucleotide excision repair sites for AFB1-induced DNA adducts within the 3D human genome organization. Our analysis revealed a heterogeneous AFB1–dG repair across four different organization levels, including chromosome territories, A/B compartments, TADs, and chromatin loops. We found that chromosomes positioned closer to the nuclear center and regions within A compartments have higher levels of nucleotide excision repair. Notably, we observed high repair activity around both TAD boundaries and loop anchors. These findings provide insights into the complex interplay between AFB1-induced DNA damage repair, transcription, and 3D genome organization, shedding light on the mechanisms underlying AFB1-induced mutagenesis.

## Graphical Abstract

**Graphical Abstract:**
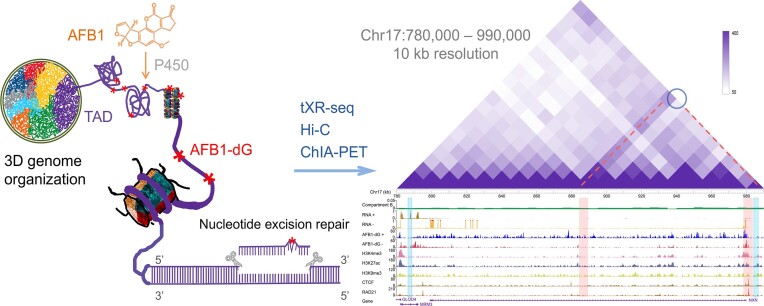

## Introduction

Aflatoxins, discovered in the early 1960s (1), are a group of mycotoxins produced by certain fungal species such as *Aspergillus flavus* and *Aspergillus parasiticus*. Aflatoxin B1 (AFB1) is the most prevalent and highly carcinogenic member of this mycotoxin family. Chronic dietary exposure to aflatoxin and hepatitis B virus infection are among the major risk factors associated with hepatocellular carcinoma (HCC), which is the third leading cause of global cancer-related fatalities (2). Aflatoxin initiates hepatocarcinogenesis through genotoxic processes, including metabolic activation to an epoxide, formation of the aflatoxin–DNA adducts and mutagenesis. Upon ingestion, AFB1 is absorbed and transported to the liver, where it is metabolized into AFB1-8,9-epoxide by cytochrome-P450 enzymes. This reactive epoxide intercalates into the DNA double helix and covalently bonds with the most nucleophilic N^7^ atom of deoxyguanosine to form an AFB1–N^7^–dG bulky adduct. This resulting AFB1–N^7^–dG adduct is unstable and can undergo depurination or spontaneous ring-opening hydrolysis which leads to the formation of an AFB1-formamidopyrimidine-dG (AFB1–FAPY–dG) adduct. Compared to AFB1–N^7^–dG, the ring-opened AFB1–FAPY–dG is more flexible and stable (3). Duplex DNA containing AFB1–FAPY–dG is less distorted and has a lower repair propensity than the AFB1–N^7^–dG adducted DNA, which might contribute to the higher mutagenicity of AFB1–FAPY–dG DNA adduct (4). If left unrepaired, the two types of AFB1–dG adduct can be bypassed during replication by error-prone translesion synthesis (TLS) DNA polymerases, leading to predominantly G to T transversions (5,6). A particularly striking example is the mutation hotspot at codon 249 (AGG to AGT) of the *TP53* gene, commonly observed in HCC patients from regions with high risk of dietary AFB1 exposure (7). Computational analysis of somatic mutations from HCC patients with AFB1 exposure reveals a unique mutation pattern known as COSMIC mutational signature 24, which has a strong transcriptional strand bias (8,9). Besides targeting duplex DNA, AFB1-8,9 epoxide can be hydrolyzed to AFB1 dialdehyde, which reacts with lysine's amino group in serum albumin to form protein adducts (10). Previous studies suggest that reactive oxygen species generated during AFB1 metabolism in liver cells initiate lipid peroxidation and its byproducts, acetaldehyde and crotonaldehyde, can also damage DNA by forming bulky cyclic propano-dG adducts (11,12). The sequence specificity of this type of damage formation and repair may contribute to the mutation hotspot seen at codon 249 of the *TP53* gene.

Nucleotide excision repair is a highly conserved and versatile repair pathway that removes various types of helix distorting and bulky DNA lesions, including AFB1–dG adducts, UV-induced cyclobutane pyrimidine dimers (CPDs) and pyrimidine–pyrimidone (6–4) photoproducts [(6–4)PPs], and benzo[*a*]pyrene (BaP)-induced DNA adducts (13–15). Nucleotide excision repair comprises two subpathways: global genomic repair and transcription-coupled repair (TCR) (16). While global genomic repair removes DNA damage throughout the entire genome, TCR, triggered by the stalling of elongating RNA polymerase II (RNAPII) at a DNA lesion (17), is dedicated to the faster repair of DNA lesions in the transcribed strand (TS) than in the non-transcribed strand (NTS) (18,19). The two subpathways differ only at the damage recognition step and share the same DNA repair machinery in the remaining repair processes including dual incisions bracketing the lesion, release of the excision products, gap filling, and ligation. Interestingly, even though the dual-incision mechanism is quite similar for prokaryotes (20), archaea (21) and eukaryotes (22), the pattern of dual-incision varies among different species (23–25). Nucleotide excision repair was presumed to be the primary repair pathway that can remove the AFB1–dG adducts (26,27). Recent studies indicate that base excision repair may also contribute to the removal of certain AFB1-induced lesions. Research has shown that the DNA glycosylase NEIL1 can recognize and excise AFB1–FAPY–dG adducts in a sequence-dependent manner (28,29).

Three-dimensional (3D) genome organization functions in various cellular processes, including gene regulation, DNA replication, and DNA damage and repair. Within the cell nucleus, the entire genome is organized into distinct hierarchical structures at different length scales, including chromosome territories, A/B compartments, topologically associating domains (TADs), and chromatin loops. Each chromosome occupies a specific region within the nucleus, which is termed a chromosome territory. At the megabase scale, chromosomes are divided into A and B compartments which represent active and inactive chromatin regions, respectively. TAD refers to a specific self-interacting region, in which chromatin regions interact more frequently than the neighboring regions (30). Chromatin interactions, such as enhancer-promoter interactions, are facilitated by a process known as loop extrusion. This mechanism allows distant regulatory elements, such as promoters and enhancers, to come into spatial proximity of targeted genes. In theory, higher-order chromatin organizations can regulate DNA damage distribution and repair efficiency. Meanwhile, DNA damage formation, DNA damage response, and repair events can alter the 3D genome structure (31–34). However, how the multiple levels of genomic organizations interplay with DNA damage formation and repair remain largely unexplored.

With the advent of high throughput next-generation sequencing (NGS) techniques, a variety of NGS-based methods, such as high-throughput chromosome conformation capture (Hi-C), Chromatin Interaction Analysis with Paired-End-Tag sequencing (ChIA-PET), Damage-sequencing (Damage-seq), and eXcision Repair-sequencing (XR-seq), have been invented to investigate 3D genome structures, DNA damage formation, and DNA repair (35–38). To date, genome-wide nucleotide excision repair of different DNA damaging agents, such as UV, BaP, cisplatin, oxaliplatin and 5-ethynyl-2′-deoxyuridine (EdU), have been analyzed at single-nucleotide resolution by using XR-seq in a variety of organisms (23,39–41). However, how DNA damage caused by AFB1, the most potent hepatocarcinogen, is removed by nucleotide excision repair at single-nucleotide resolution on a genome-wide scale is still unexplored. Furthermore, the distribution of AFB1–dG repair within the 3D human genome organization remains unknown.

In this study, we adapted the translesion XR-seq (tXR-seq) method (39) to map genome-wide AFB1–dG repair and compared it with repair data obtained from conventional CPD/(6–4)PP XR-seq. Our results showed that AFB1–dG adducts are mainly removed by TCR. To gain a comprehensive understanding of the AFB1–dG repair, we then integrated datasets from Hi-C, ChIA-PET, DNA fluorescence *in situ* hybridization (FISH) and tXR-seq to investigate the AFB1–dG repair across four different organization levels, including chromosome territories, A/B compartments, TADs, and chromatin loops. Our analysis revealed a heterogeneous AFB1–dG repair landscape within the 3D genome organization. These findings provide insights into the complex interplay between nucleotide excision repair, transcription and 3D genome organization, paving the way for our understanding of AFB1-induced mutagenesis.

## Materials and methods

### Cell line and culture conditions

The human HepG2 cell line was purchased from the American Type Culture Collection (ATCC). AFB1 (Cat. No. A6636) was obtained from MilliporeSigma. HepG2 cells were cultured in Dulbecco's Modified Eagle Medium (DMEM) supplemented with 10% FBS at 37°C in a 5% CO_2_ humidified chamber.

### UVC irradiation and AFB1 treatment

HepG2 cells were cultured until they reached approximately 80% confluence, after which they were subjected to UVC irradiation (20 J/m^2^) using a GE germicidal lamp emitting primarily 254-nm UV light. Following UV exposure, the cells were cultured at 37°C for 0.5 h before conducting an *in vivo* excision assay and for 4 h before performing CPD/(6–4)PP XR-seq. To ensure a more uniform exposure to AFB1 while using a smaller volume of this carcinogen, we treated HepG2 cells with AFB1 in suspension. Briefly, an AFB1 stock solution (4 mM) was added to the HepG2 cell culture medium in a cell culture flask to reach a final concentration of 40 μM, which resulted in approximately 70% cell viability (42). The expression levels of CYP3A4, CYP1A2 and CYP3A5, which are capable of bioactivating AFB1, in HepG2 cells are generally lower than in primary human hepatocytes (43,44). To facilitate the bioactivation of AFB1 during the treatment, we added the rat liver microsomes (Sigma, Cat. No. M9066) to a concentration of 0.5 mg/ml. Then, the cells (approximately 4 × 10^7^) were cultured in 50 ml DMEM medium for 4 h before performing tXR-seq, with gentle shaking every 30 min to ensure cell suspension in the medium.

### Antibodies, TLS DNA polymerase and oligonucleotides

The following antibodies were used in this study: anti-XPB (Santa Cruz, sc293), anti-XPG antibody (Santa Cruz, sc13563), rabbit anti-mouse IgG (Abcam, ab46540), anti-AFB1 monoclonal antibody (clone 6A10) (Thermo Fisher, MA1-16885) and anti-AFB1 (clone AFA-1) (Abcam, ab1017). Sulfolobus DNA polymerase IV (Dpo4) (Cat. No. M0327) and DNA polymerase ζ (Cat. No. 51) were purchased from NEB and Enzymax, respectively. Oligonucleotides used for adaptor ligation and PCR amplification of the sequencing library were the same as described previously (23).

### *In vivo* excision assay

The *in vivo* excision assay procedure followed the general approach described previously (45). Briefly, HepG2 cells (from two 80% confluent 150 mm petri dishes) were either treated with 40 μM AFB1 and 0.5 mg/ml rat liver microsomes for 4 h, exposed to UVC irradiation (20 J/m^2^) and allowed to repair for 0.5 h, or left untreated as a non-damaged control. Cells were then lysed using the Hirt lysis method. After precipitation of high molecular weight DNA, the supernatant was subjected to immunoprecipitation (IP) with anti-XPB (4 μl) and anti-XPG (4 μl) antibodies to capture excision products released during nucleotide excision repair. The isolated excision products were purified, 3′ end-labeled with 1 μCi ^32^P-Cordycepin by 1 μl terminal deoxynucleotidyl transferase (NEB, Cat. No. M0315L) at 37°C for 1 h and resolved on a 10% denaturing polyacrylamide sequencing gel. The gel was then dried, exposed to a phosphor screen overnight, and scanned using a Typhoon FLA 9500 biomolecular imager. This method allows for the visualization and comparison of excision products from AFB1 or UVC treatment, as well as the non-damaged control.

### Library construction for CPD/(6–4)PP XR-seq and AFB1–dG tXR-seq

CPD/(6–4)PP XR-seq libraries were prepared as described in our previous study (23). AFB1–dG tXR-seq libraries were prepared following our previous study's protocol (39) with below modifications: After TFIIH/XPG IP, the excision products were treated with carbonate-bicarbonate buffer (10 mM Tris–Cl pH 7.5, 1 mM EDTA, 1% SDS, 30 mM NaHCO_3_ and 15 mM Na_2_CO_3_, pH 9.6) at 37°C for 2 h to convert the AFB1–N^7^–dG to the ring-opened AFB1–FAPY–dG adducts (46–48). Following adaptor ligation, the adaptor-ligated excision products were further purified by IP with anti-AFB1 monoclonal antibody (clone 6A10). To bypass the AFB1–FAPY–dG adduct, a 20 μl reaction mixture was prepared containing 4 μl of 5 × Dpo4 reaction buffer, 3 μl of RPIn Reverse primer, 10 μl of adaptor-ligated excision products, and 3 μl of Dpo4 enzyme. The primer extension reaction was carried out at 55°C for 10 min. After purification of the primer extension products by phenol-chloroform extraction, Kapa Hotstart Readymix (Kapa Biosystems, Cat. No. KK2602) was used for amplifying the library. All libraries from CPD/(6–4)PP XR-seq and AFB1–dG tXR-seq were sequenced on the Illumina HiSeq 2500 platform.

### Data processing and visualization

Two biological replicates of CPD/(6–4)PP XR-seq and AFB1 tXR-seq were used for the analysis. The adaptors were trimmed by using BBduk and reads longer than 50 mers were filtered out for analysis. PCR duplicates were removed by the FASTX-Toolkit. Refined reads were aligned to the human reference genome GRCh38 by using the bowtie tool (49) with arguments: -x -q –nomaqround -m 4 -v 3 –tryhard –strata –best -p 4 –seed = 123. The aligned reads were normalized with the sequencing depth and visualized with Integrative Genomics Viewer (50).

### Data collection

XR-seq datasets used for length distribution analysis were downloaded from the Gene Expression Omnibus with accession numbers: GSE97675 (BPDE), GSM6222398 (EdU), GSE82213 (cisplatin), GSE67941 [CPD and (6–4)PP]. The processed *in-situ* Hi-C datasets from the HepG2 cell line were downloaded from the ENCODE consortium (ID: ENCSR194SRI). The processed CTCF-mediated ChIA-PET data from HepG2 cells was downloaded from ENCODE (ID: ENCSR411IVB). RNA-seq, lamin A/C ChIP-seq and lamin B1 ChIP-seq data from HepG2 cells were obtained from ENCODE (GEO: GSE119631). ChIP-seq data for histone marks (H3K4me3, H3K9me3, H3K27ac) from HepG2 cells were obtained from ENCODE (ID: ENCSR134DWG). Rad21 ChIP-seq data from HepG2 cells were downloaded from ENCODE (ID: ENCSR000EEG).

### Length distribution and nucleotide frequency analysis

The length distributions for all reads and single nucleotide frequencies for selected excision products from tXR-seq and XR-seq were calculated using in-house custom scripts.

### Average repair profile analysis for CPD, (6–4)PP and AFB1–dG

The CPD and (6–4)PP repair levels for each selected region were counted and normalized to RPKM (Reads Per Kilobase per Million mapped reads). As AFB1-8,9-epoxide preferentially binds to the N^7^ position of guanine, the GC content within each selected region, such as chromosome, A/B compartment, TAD, loop, gene, TS and NTS, may cause bias in our analysis. We then used the total number of Gs in the selected region for the normalization, which we termed ‘RPKGM’ shown in the following equation:


\begin{equation*}{\mathrm{RPKGM}} = \frac{{{\mathrm{Number\;of\;reads\;mapped\;to\;the\;region*}}{{10}^9}}}{{{\mathrm{Total\;number\;of\;mapped\;reads*Number\;of\;Gs}}}}\end{equation*}

For analysis of the effect of transcription on CPD, (6–4)PP and AFB1–dG repair, the gene list file of the GRCh38 genome assembly was downloaded from the USCS genome browser. To focus the analysis on highly expressed and long genes, genes with an expression score greater than 300 and a length greater than 3 kb were selected. Among those genes, those with another neighboring transcript within the ±6 kb vicinity were removed. The selected 20441 genes were divided into 100 bins and their ±6 kb regions were divided into 50 bins. Then, they were intersected with the mapped read counts of both positive and negative strands to get the read counts for both TS and NTS. The numbers of reads in each bin were calculated and normalized to get RPKM or RPKGM values.

For the chromosome wise analysis of AFB1–dG repair, we calculated the total number of Gs for each chromosome from human reference genome GRCh38 and intersected each chromosome with AFB1–dG tXR-seq mapped reads to get the total number of mapped reads. Then, we obtained the AFB1–dG repair level based on the above RPKGM equation. For the A/B compartments analysis, we downloaded A/B compartments (ID: ENCFF091UMY) of the HepG2 cells from the ENCODE. We separated each compartment into three categories: A, B and conserved compartments, based on the Principal Component Analysis value. Bedtools was used to intersect the A/B compartments with AFB1–dG tXR-seq mapped reads of both TS and NTS to calculate the repair level in A/B compartments.

In the analysis of CPD, (6–4)PP, and AFB1–dG repair within TADs, processed Hi-C data (file ID: ENCFF018XKF) from the HepG2 cell line was used for the analysis. Overlapping TADs were removed from further analysis, and the resulting nonoverlapping TADs (4311 in total) were intersected with mapped reads from CPD/(6–4)PP XR-seq and AFB1–dG tXR-seq using bedtools (51). Read counts were calculated at each TAD and were normalized by either RPKM or RPKGM equation. Additionally, the selected TADs were divided into quartiles (*Q1 to Q4*) based on the TAD corner score and intersected with the mapped reads to calculate the number of read counts in the TAD boundaries and intra-TAD regions for each quartile. For TAD boundary analysis, we defined the boundary of each TAD by extending the start and end to four different window sizes ±1, ±5, ±10 and ±50 kb, and created the new sub-starts and sub-ends of boundary regions. The intra-TAD regions were defined as follows: we initially calculated the center (mid-point) of each TAD, and then extended these center points on both sides using the same methodology as that employed for extending the window sizes at TAD boundaries.

### Statistical analysis and visualization

Statistical calculations such as the Wilcoxon test was performed using R-Packages. The ggplot2 package was used for visualization and plotting in R-Studio. Hi-C heatmaps were visualized by WashU Epigenome Browser (http://epigenomegateway.wustl.edu/browser/).

## Results

### Adaptation of tXR-seq for genome-wide mapping nucleotide excision repair of AFB1-induced DNA damage

**Figure 1.**
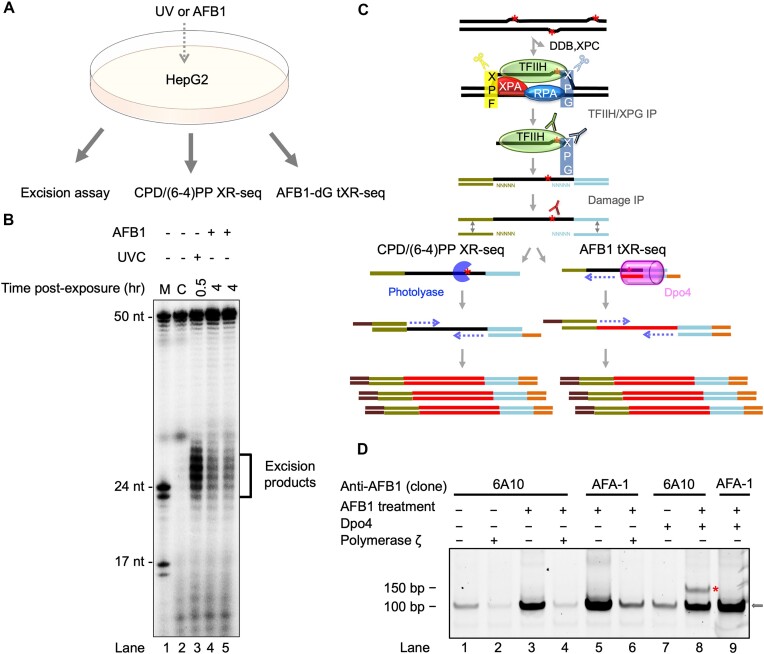
Experimental design and adaptation of tXR-seq for mapping nucleotide excision repair of AFB1–dG adducts. (**A**) Overview of the experimental design for *in vivo* excision assay, CPD/(6–4)PP XR-seq (4 h post-exposure) and AFB1–dG tXR-seq (4 h post-exposure). (**B**) Detection of excision products by excision assay after UVC (20 J/m^2^) or AFB1 (40 μM) treatment in HepG2 cells. Excision products from one-quarter of a 150 mm petri dish of UVC-treated HepG2 cells (0.5 h post-exposure) were loaded into the gel, while for AFB1-treated HepG2 cells (4 h post-exposure), excision products from two 150 mm petri dishes were used in the excision assay. M, DNA size marker; C, non-damaged control. (**C**) Sequencing library construction workflow for CPD/(6–4)PP XR-seq and AFB1–dG tXR-seq. Red asterisk indicates DNA bulky adduct. Excision products, released during nucleotide excision repair, are precipitated with TFIIH/XPG antibodies, extracted and ligated to adaptors. Then the adaptor-containing oligomers are precipitated with damage IP. Photolyases are used for the reversal of UV damage for CPD/(6–4)PP XR-seq. For AFB1–dG tXR-seq, Dpo4 (pink) is the sulfolobus DNA polymerase IV, a Y-family DNA polymerase known for its ability to bypass various DNA lesions. It is used for bypassing the AFB1–dG damage during the primer extension before the PCR amplification. (**D**) Optimization of AFB1–dG tXR-seq library preparation. Lanes show results using different anti-AFB1 antibodies (6A10 and AFA-1) and translesion synthesis DNA polymerases (Dpo4 and polymerase ζ). Lanes 1, 2, and 7 are non-damaged controls. Libraries were analyzed by 10% native polyacrylamide gel electrophoresis. Red star marks PCR products containing inserts; black arrow indicates adapter dimers.

During nucleotide excision repair, excision products containing the damage are released in complex with TFIIH-XPG. In the conventional XR-seq method (23), these excision products are captured, sequenced, and mapped to the reference genome. Before library amplification by PCR, the DNA damage within the excision products must be removed either enzymatically or chemically. In contrast, tXR-seq employs appropriate translesion DNA synthesis (TLS) polymerases to bypass the damage before PCR amplification, making it applicable for essentially all types of DNA lesions removed by nucleotide excision repair (39). As the quantity of excision products is crucial for the success of tXR-seq, we performed an *in vivo* excision assay using HepG2 cells prior to conducting the CPD/(6–4)PP XR-seq, AFB1–dG tXR-seq (Figure 1A–D). As shown in Figure 1B, although the yield is lower than that in UVC-treated (20 J/m^2^) HepG2 cells, the excision products are detectable when HepG2 cells are treated with AFB1 (40 μM) for 4 h. Of note, in the non-damaged control sample (Figure 1B, lane 2), there is no signal from excision products (20- to 30-mers), and therefore, no library from the non-damaged control sample could be sequenced for further analysis. This indicates that the reads obtained from the CPD/(6–4)PP XR-seq and AFB1–dG tXR-seq are excision products exclusively released during nucleotide excision repair. The two-step IP procedure effectively excludes most non-specific binding of genomic-DNA fragments, resulting in nearly zero background for both XR-seq and tXR-seq (Figure 1C).

In our AFB1–dG tXR-seq procedure, we first isolated DNA excision products containing AFB1-DNA adducts using IP with TFIIH/XPG antibodies. We then chemically converted the AFB1–N^7^–dG adducts to the more stable ring-opened AFB1–FAPY–dG adducts. The unstable AFB1–N^7^–dG adducts are quantitatively abundant within 24 h after AFB1 treatment (52) and the conversion avoids their depurination. If depurination occurs, it would result in an abasic site rather than an AFB1–FAPY–dG bulky adduct. Therefore, the anti-AFB1 antibody, which specifically recognizes the AFB1–FAPY–dG adduct, would not be able to bind to it (46,48). We then determined the optimal AFB1 antibody and TLS polymerase that could capture the excision products and bypass the converted AFB1–FAPY–dG lesion, respectively. Gel electrophoresis of the libraries, generated by PCR amplification of the AFB1–FAPY–dG containing excision products, indicated that Dpo4 could bypass the AFB1–FAPY–dG adduct and be used for our tXR-seq method (Figure 1D and Supplementary Figure S1A).

After sequencing, the nucleotide frequencies of the excision products from the raw reads were analyzed. As shown in Supplementary Figure S1C, D, guanines (Gs) are enriched at positions 17–22 for 25-mer excision products. Since the Y-family DNA polymerase Dpo4 introduces mainly G to T transversions during lesion bypass, the raw sequencing reads from our AFB1–dG tXR-seq may contain these mutations. After mapping the raw reads to the human reference genome, we extracted the mapped reads sequences from the reference genome and compared them with our raw sequenced reads. Our comparison showed G frequencies at positions 17–22 for the 25-mer excision products in the extracted reads shown in Supplementary Figure S1D are higher than in the raw reads (Supplementary Figure S1C). The enrichment of Gs at position 19 can be seen from 24-mers to 27-mers (Supplementary Figure S2). As expected, TT and TC are enriched at positions 17–22 for the 25-mer excision products from CPD XR-seq and (6–4)PP XR-seq, respectively (Supplementary Figure S1E and F). Further analysis of the frequency change after bypass confirmed the predominant G to T transversions introduced by the error-prone Dpo4 (Supplementary Figure S3). Interestingly, we observed a distinct excision product length distribution, with the predominant length being only 25 nt, in contrast to excision products containing other DNA damaging agents-induced adducts where 26-mers are most frequent (Supplementary Figure S4) (23,39–41).

### Effect of transcription on AFB1–dG repair

**Figure 2.**
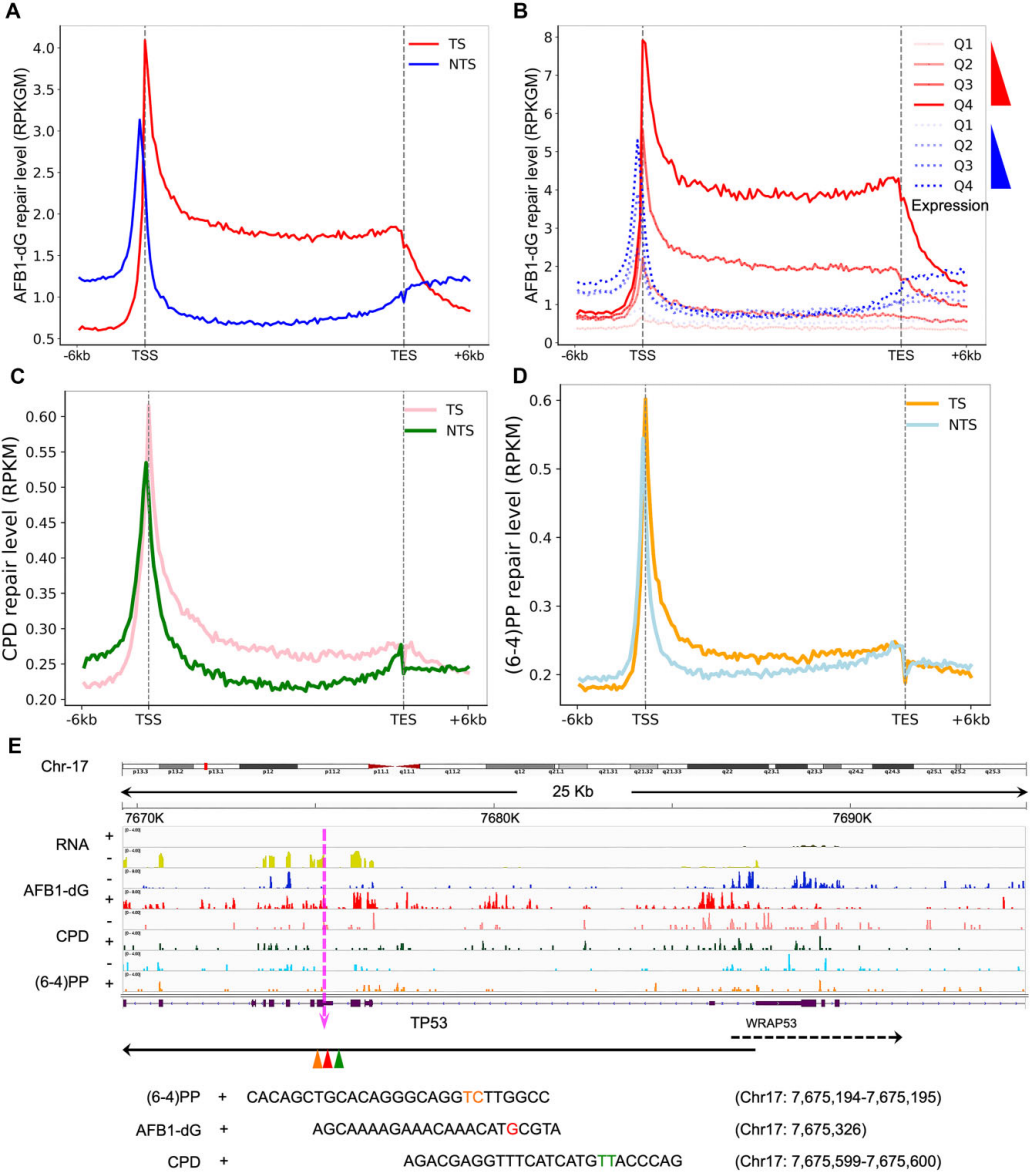
Effect of transcription on AFB1–dG, CPD and (6–4)PP repair and visualization of their repair signals on *TP53* and *WRAP53* genes in chromosome 17. (**A**) AFB1–dG repair profiles around the transcription start site (TSS) and transcription end site (TES) of 20441 selected genes at 4 h post-exposure in HepG2 cells. TS and NTS are shown in red and blue, respectively. TS, transcribed strand; NTS, nontranscribed strand. (**B**) AFB1–dG repair profiles over 20441 selected genes separated into quartiles based on expression score. Q1 is the lowest expression quartile and Q4 is the highest one. (**C**) CPD repair profiles around the TSS and TES of 20441 selected genes at 4 h post-exposure in HepG2 cells. TS and NTS are shown in pink and green, respectively. (**D**) (6–4)PP repair profiles around the TSS and TES of 20441 selected genes at 4 h post-exposure in HepG2 cells. TS and NTS are shown in orange and light blue, respectively. **(E)** Screenshot of AFB1–dG, CPD, and (6–4)PP repair signals on *TP53* and *WRAP53* genes in chromosome 17. ‘+’ in RNA denotes genes transcribed from left to right, while ‘–’ indicates right-to-left transcription. In CPD/(6–4)PP XR-seq and AFB1 tXR-seq, ‘+’ signifies plus-strand DNA (5′ to 3′ direction), and ‘–’ denotes minus-strand DNA (3′ to 5′ direction). Three representative reads from CPD and (6–4)PP XR-seq, and AFB1–dG tXR-seq are shown. The pink dashed line indicates the zoomed-in region in *TP53* gene. Orange TC (chr17: 7675194–7675194), red G (chr17: 7675326), and green TT (chr17: 7675599–7675600) indicate the positions of the respective damages in the *TP53* gene.

Nucleotide excision repair of DNA lesions is regulated by various factors such as chromatin states, transcription factor binding, DNA replication, transcription, and circadian clock (53–58). The effect of transcription on nucleotide excision repair has been extensively studied since the discovery of TCR in human cells (59). As aforementioned, AFB1–dG adducts are repair-resistant because of their intercalated conformations in the DNA double helix. Thus, it is conceivable that AFB1–dG adducts are mainly removed through TCR. We aligned the AFB1–dG tXR-seq reads onto the human reference genome and generated the genome-wide repair map of AFB1–dG adducts. The correlation analysis between the two biological replicates showed high reproducibility (minimum Pearson *r* = 0.91, Supplementary Figure S5). Our analysis of the AFB1–dG repair profiles for TS and NTS revealed that TS is preferentially repaired over NTS (Figure 2A). As expected, AFB1–dG repair for both TS and NTS peaks around transcription start sites (TSS) and dramatically declines towards transcription end sites (TES). We next proceeded to investigate the impact of gene expression on AFB1–dG repair. Based on the expression level, genes were divided into four groups, ranging from Q1 (bottom 25%) to Q4 (top 25%). Then, the average strand-specific repair profiles for each of the four groups were plotted (Figure 2B). As can be seen, higher gene expression levels are associated with more efficient AFB1–dG repair on both TS and NTS. It has been known that the removal of CPDs is primarily dependent on the TCR pathway, while (6–4)PPs are mainly removed by the global genomic repair pathway in a more rapid and efficient way (23,58,60,61). Indeed, the TS/NTS repair ratio for CPD is much higher than that for (6–4)PP (Figure 2C and D). However, the TS/NTS repair ratio for CPD is lower than that for AFB1–dG (Figure 2A). The trend of AFB1–dG repair around the TSS and TES is also in general agreement with previously identified CPD repair in human NHF1 and GM12878 cells (23,39). Interestingly, Figure 2D shows some degree of preferential repair of (6–4)PPs on the TS. This can be due to two factors in our experimental design and analysis. To enable a direct comparison of repair patterns across CPDs, (6–4)PPs, and AFB1–dG lesions, we chose a common time point of 4 h post-treatment for all analyses. Given that most (6–4)PPs are removed by global genome repair within this timeframe, the remaining (6–4)PPs on the TS of actively transcribed genes are predominantly removed by TCR after 4 h. The other factor is that we focused on a subset of highly expressed and longer genes. Specifically, we selected only 20441 genes with high expression levels (expression score > 300) and substantial length (>3 kb). This subset is enriched for highly transcribed genes where TCR effects are more pronounced. Of note, we compared the TCR of CPDs, (6–4)PPs and AFB1–dG in the same cell line and the same time point because TCR efficiency can vary between cell types due to differences in protein levels and activity of repair complexes like CRL4–DDB2 (62).

To visualize the interplay between nucleotide excision repair and transcription throughout the whole genome, we used datasets from RNA-seq, AFB1–dG tXR-seq and CPD/(6–4)PP XR-seq to generate a high-resolution repair map at the single nucleotide level. Figure 2E shows the transcription and repair maps of the *TP53* tumor suppressor gene on chromosome 17. It is evident that the AFB1–dG repair level on the TS of *TP53* is much higher than on the NTS. Similarly, CPD repair on the TS shows higher levels compared with the NTS. In contrast, the repair levels for (6–4)PPs on the TS and NTS of *TP53* are more comparable. Specifically, AFB1–dG and CPD repair on the TS peaks within *TP53* exons, coinciding with RNA-seq signals. Sharp repair peaks on the NTS may be due to factors such as sequence context, chromatin structure, and histone modifications within these regions. Three excision products from (6–4)PP XR-seq, AFB1–dG tXR-seq, and CPD XR-seq can be unambiguously assigned to specific TC, G and TT damage, respectively. Additionally, this preferential repair on the TS extends to the adjacent *WRAP53* gene, which is transcribed in the opposite direction.

### Distribution of AFB1–dG repair within the human 3D genome organization

**Figure 3.**
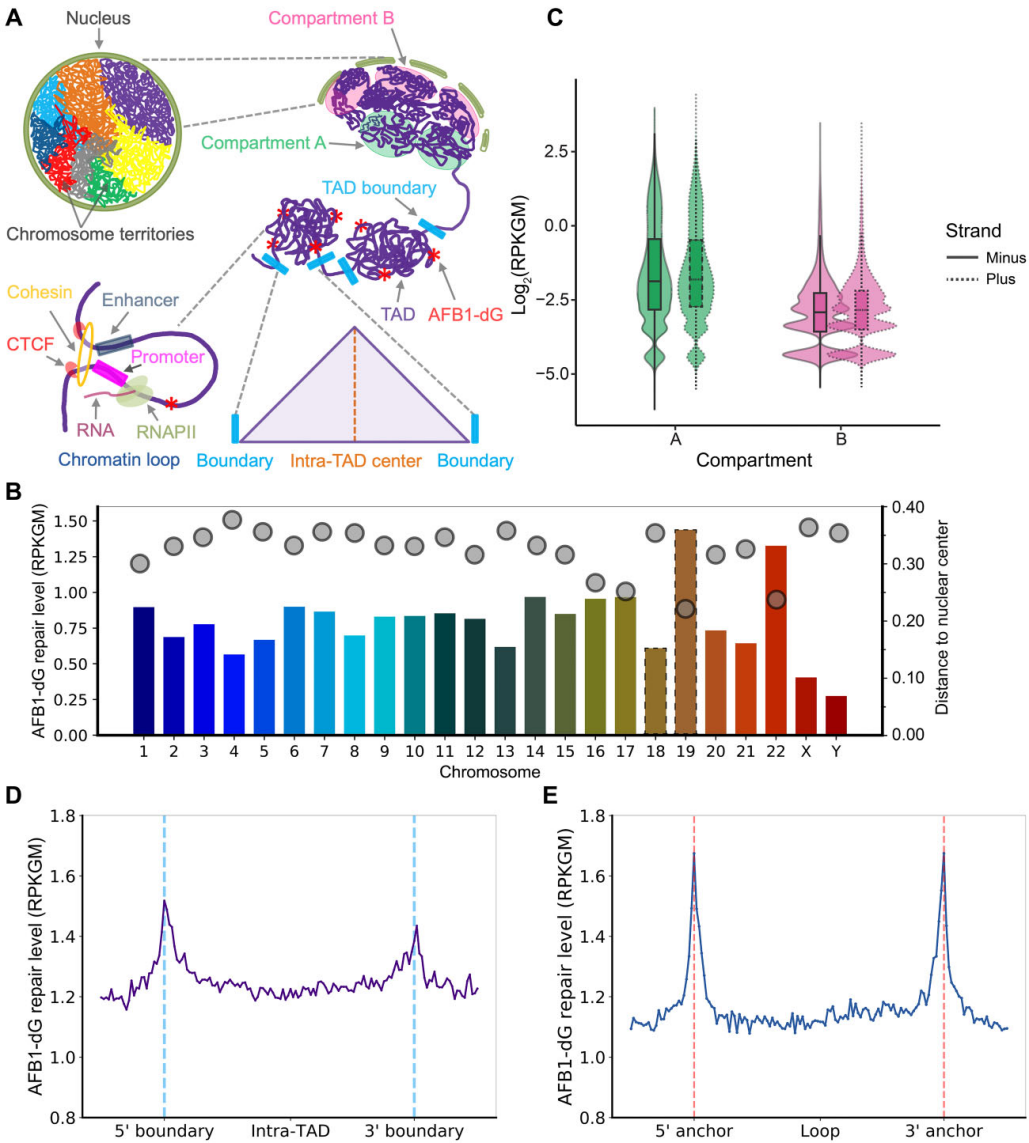
Distribution of AFB1–dG repair within the human 3D genome. (**A**) The hierarchical 3D genome is structured at four levels: chromosome territories, A/B compartments, TADs, and chromatin loops. AFB1–dG DNA adducts within TADs are marked by red stars. (**B**) Chromosome wise distribution of AFB1–dG repair and chromosome position within the human nucleus. The AFB1–dG repair level on each chromosome is depicted by bar plot. Repair values for chromosomes 2, 16, 17 and 20 are normalized according to their chromosome numbers. HSA-18 and -19 are highlighted by a dashed line. Gray dots indicate the distance to the nuclear center for each chromosome. (**C**) Violin plots illustrate AFB1–dG repair levels in A (green) and B (pink) compartments. Plus and minus strands are represented by solid and dashed lines, respectively. The solid band within the box represents the median. (**D**) Average AFB1–dG repair level over 4311 nonoverlapping TADs. 5′ and 3′ boundaries are denoted by blue dashed lines. (**E**) Average AFB1–dG repair level across 2989 nonoverlapping chromatin loops that are less than 200 kb. 5′ and 3′ anchors are denoted by red dashed lines.

In the human cell, the 23 pairs of chromosomes are packaged into a 5–10 μm diameter nucleus through a hierarchy of compaction. The spatial structures of the human genome are organized at four levels: chromosome territories, A/B compartments, TADs, and chromatin loops (Figure 3A). Unlike *in vitro* repair assays conducted with linear DNA, the cellular DNA repair machinery faces the challenge of surmounting the structural barriers created by DNA packaging. Various factors, including transcription, DNA replication, histone modifications, circadian clock, and regulatory protein binding, have been found to interplay with DNA damage and repair (39,55,63–67). To date, the distribution of AFB1–dG repair within the 3D human genome organization remains unknown.

To explore the correlation between chromosome territory positioning and nucleotide excision repair, we calculated the AFB1–dG repair levels on the 23 chromosomes and compared them with the DNA FISH data (68). As shown in Figure 3B, our results indicated that AFB1–dG repair exhibits a non-uniform distribution across all the chromosomes. Notably, repair levels tend to be higher for chromosomes positioned closer to the nuclear center. This trend is especially evident in chromosomes 18 and 19 (HSA-18 and -19), which are situated in the nuclear periphery and center, respectively. The repair level of gene-rich HSA-19 is approximately 3-fold higher than that of gene-poor HSA-18.

The A compartments are localized within the interior of the nucleus and represent regions of open and expression-active chromatin marked by histone modifications such as H3K4me3 and H3K27ac. In contrast, the B compartments lie at the nuclear periphery and are associated with closed and expression-inactive chromatin, marked by repressive histone markers like H3K9me3 (Figure 3A). To investigate the distribution of AFB1–dG repair within A/B compartments, we analyzed the average repair levels in all the A/B compartments identified from *in situ* Hi-C experiments in HepG2 cells (69). As aforementioned, AFB1–dG adducts are repaired mainly through TCR subpathway, it is plausible to predict that AFB1–dG repair activity in the A compartments is higher than that in B compartments. Indeed, the average AFB1–dG repair levels on the two DNA strands in A compartments surpass those observed in B compartments (Figure 3C).

To determine the distribution of AFB1–dG repair within TADs, we generated the average AFB1–dG tXR-seq signal relative to TADs defined with Hi-C data from HepG2 cells. Remarkably, our findings reveal a notable enrichment of average AFB1–dG repair levels around both the 5′ and 3′ boundaries of non-overlapping TADs (Figure 3D and Supplementary Figure S6A). It's noteworthy that the repair levels in the intra-TAD center are comparable to inter-TAD regions. Given that both TADs and chromatin loops are established through the loop extrusion process and dependent on the binding of CTCF and cohesion, it is reasonable to anticipate that chromatin loops have a similar repair pattern to that observed within TADs. Our analysis demonstrates a significant enrichment of average AFB1–dG repair signals around the two anchors of non-overlapping chromatin loops, all of which are less than 200 kb in size (Figure 3E).

**Figure 4.**
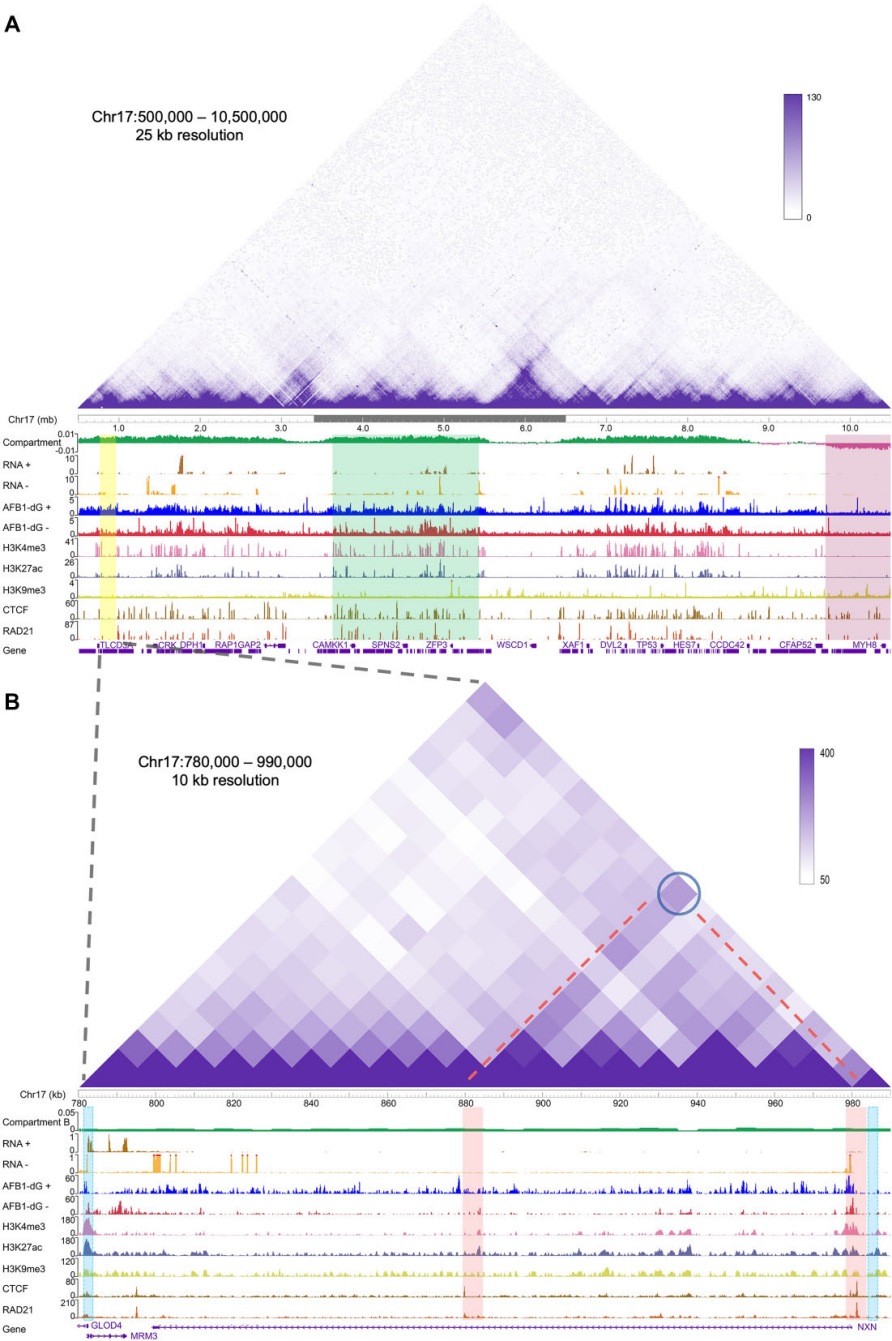
Screenshots of AFB1–dG repair for chromosome 17 across A/B compartments, TADs, and chromatin loops. (**A**) Hi-C heatmap at 25-kb resolution covering a 10 mb region on chromosome 17. A and B compartments are shown in green and pink, respectively. AFB1–dG tXR-seq data and public RNA-seq data, ChIP-seq data for histone marks (H3K4me3, H3K27ac, and H3K9me3), CTCF and RAD21 are shown below the heatmap. The selected region is highlighted in yellow. (**B**) Zoomed view of AFB1–dG repair over the highlighted 210 kb region in Figure 4A. TAD and anchor boundaries are highlighted in light blue and red, respectively. The representative chromatin loop is indicated by a dark blue circle and red dashed line.

The distribution of AFB1–dG repair within A/B compartments, TADs, and chromatin loops is further illustrated in Figure 4. In Figure 4A, the Hi-C heatmap of chromosome 17 at a resolution of 25 kb is displayed using the 3D Genome Browser. Additionally, data from AFB1–dG tXR-seq, Hi-C, RNA-seq and ChIP-seq for CTCF, the cohesion subunit RAD21, and three histone marks (H3K4me3, H3K27ac, and H3K9me3) are visualized using the WashU Epigenome Browser. As apparent from the data, AFB1–dG repair signals are notably higher within the A compartment (shaded in green) compared to the B compartment (shaded in pink). Meanwhile, ChIP-seq signals for active histone marks (H3K4me3 and H3K27ac) within the A compartment significantly surpass those in the B compartment. Zooming into the yellow-shaded region in Figure 4A, we identified a TAD spanning 200 kb, containing the NXN gene, where AFB1–dG repair signals are enriched around the 5′ and 3′ boundaries (shaded in light blue). Within this TAD, a loop is evident. This loop features two anchor regions highlighted in red (Figure 4B). Notably, AFB1–dG repair signals are higher around these anchor regions, mirroring the heightened ChIP-seq signals for CTCF and RAD21. We observed that AFB1–dG repair on the TS of the NXN gene substantially exceeds that on the NTS, indicating the substantial impact of transcription on the repair process.

Altogether, our findings reveal a heterogenous distribution of nucleotide excision repair sites for AFB1-induced DNA adducts within the 3D human genome organization, suggesting the hierarchical levels of 3D genome organization may interplay with the repair process. Evidently, AFB1–dG adducts residing on chromosomes proximal to the nuclear center are prioritized for repair. The A compartments, associated with an open chromatin landscape, are preferentially targeted for repair, in contrast to the relatively less accessible B compartments. Our results indicate high repair levels at both the boundaries of TADs and the anchor regions of chromatin loops. These findings underscore the intricate interplay between 3D genome organization and the nucleotide excision repair machinery in maintaining genomic integrity.

### High levels of AFB1–dG repair within TAD boundaries and loop anchors

**Figure 5.**
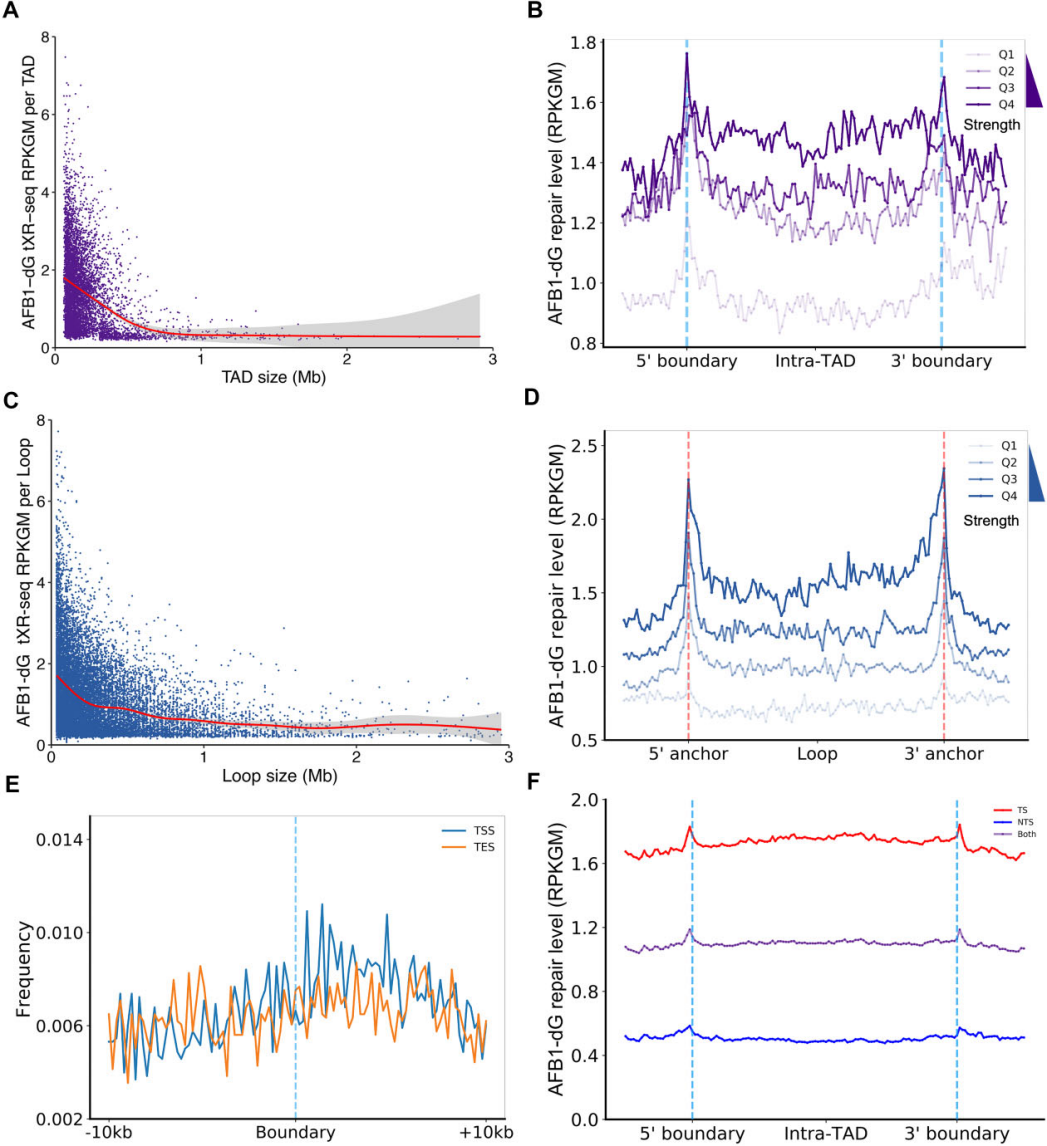
High levels of AFB1–dG repair within TAD boundaries and loop anchors. (**A**) A scatter plot illustrates AFB1–dG repair levels per TAD relative to TAD size. There are a total of 6183 TADs included in this analysis. (**B**) AFB1–dG repair profiles are displayed across 4311 nonoverlapping TADs, categorized into quartiles based on TAD corner score, ranging from Q1 (lowest score) to Q4 (highest score). (**C**) A scatter plot shows AFB1–dG repair levels per chromatin loop relative to loop size. The total number of chromatin loops shown here is 14445. (**D**) AFB1–dG repair profiles are shown across 2989 nonoverlapping chromatin loops (length < 200 kb), separated into quartiles based on loop strength scores, ranging from Q1 (lowest strength) to Q4 (highest strength). (**E**) Frequency distributions of TSS and TES surrounding boundary regions of 4311 nonoverlapping TADs. (**F**) AFB1–dG repair profiles for TS and NTS over 4311 nonoverlapping TADs.

TADs, characterized by an elevated contact frequency within them, are conserved not only across diverse cell types but also between different species (70). These structural units vary in size, ranging from 40 kb to 3 Mb (71). Many TADs show ‘corner peaks’ in their Hi-C heatmaps, indicating the presence of chromatin loops. These ‘corner peaks’ mark the anchor points of these chromatin loops. To investigate the correlation between TAD size and AFB1–dG repair, we conducted an analysis of the AFB1–dG repair levels within non-overlapping TADs as a function of TAD size. As illustrated in Figure 5A, our findings reveal a notable decrease in repair with increasing TAD size. In the Hi-C data we utilized, the arrowhead algorithm was used to identify the corner of a contact domain, thereby enabling the localization of TAD boundaries (71). The corner score, which represents the likelihood of a pixel is at the corner of a contact domain, is employed in this method. The higher corner score values indicate a greater probability of being at the domain's corner. To examine the correlation between TAD corner score and AFB1–dG repair, we divided TADs into quartiles based on their corner score and plotted the average repair levels for each quartile (Figure 5B). We observed a positive correlation between TAD corner score and AFB1–dG repair levels. For the highest corner score quartile (Q4), the repair level in the intra-TAD region is much higher than that in the inter-TAD region. Conversely, within the lowest corner score quartile (Q1), the reverse pattern is observed, suggesting that a TAD with high corner score undergoes rapid repair process. We then assessed the correlation between AFB1–dG repair and the size and strength of the chromatin loop by using a similar analytical approach. We found that loop size negatively correlates with repair, while loop strength is positively associated with repair, which mirrors the trends observed within TADs (Figure 5C and D, and Supplementary Figure S6B). Subsequently, we extended our analysis to evaluate the association between AFB1–dG repair and the size and strength of loop identified from CTCF ChIA-PET data. Our findings revealed a similar trend, with loop size negatively correlating with repair (Supplementary Figure S6C). However, we observed that the strength of loops identified from the CTCF ChIA-PET experiment did not exhibit a noticeable correlation with AFB1–dG repair (Supplementary Figure S6D).

Our results revealed elevated levels of AFB1–dG repair within regions encompassing TAD boundaries and loop anchors. We hypothesized that this phenomenon may be attributed to the elevated levels of transcription activity within those regions. We examined the distribution of TSS and TES around TAD boundaries and found an enrichment of TSS frequencies within boundary regions (Figure 5E). Notably, TSS signals displayed a decrease in the center of both 5′ and 3′ boundaries. Around the 5′ boundary region, TSS frequency was notably high, while TSS signals exhibited an increase only downstream of the 3′ boundary (Supplementary Figure S6, E and F). Further analysis of the average repair profiles for the TS and NTS across non-overlapping TADs revealed that TS repair levels were approximately four-fold higher than NTS repair levels (Figure 5F). NTS repair levels were also elevated within both the 5′ and 3′ boundary regions, suggesting that factors beyond transcription-associated factors, such as histone modifications and chromatin-binding proteins, may contribute to the elevated repair observed on the NTS within boundary regions.

To further investigate the relationship between different types of DNA damage repair and genomic structural features, we extended our analysis to include CPD and (6–4)PP repair (Supplementary Figure S7). Our results indicate that CPD repair levels are higher within the regions flanking both TAD boundaries and loop anchors (Supplementary Figure S7A and B). While (6–4)PP repair levels show peaks around TAD boundaries and loop anchors (Supplementary Figure S7C and D), they are not as high as CPD repair levels, particularly in the vicinity of TAD boundaries. This difference may reflect the contribution of TCR to CPD repair in these regions, as well as differences in damage recognition and repair kinetics between CPDs and (6–4)PPs. Previous studies showed that these regions flanking CTCF binding sites, often referred to as nucleosome-depleted regions or nucleosome-free regions, are typically associated with higher nucleotide excision repair activity (72,73). The absence of nucleosomes in these areas likely facilitates greater access for both transcription factors and nucleotide excision repair proteins. Additionally, these more exposed regions may be more susceptible to DNA damaging agents, potentially resulting in higher damage levels, which could also contribute to the observed high repair activity. The elevated levels of transcription activity, high accessibility of repair proteins, and increased levels of initial DNA damage formation may contribute to the observed high repair activity around TAD boundaries and loop anchors.

## Discussion

In liver cells, AFB1 is metabolized to AFB1-8,9-epoxide which intercalates into the DNA double helix and forms AFB1–dG bulky adducts (AFB1–N^7^–dG and AFB1–FAPY–dG). Meanwhile, the reactive oxygen species generated during AFB1 metabolism lead to lipid peroxidation and their byproducts, such as acetaldehyde and crotonaldehyde, can form bulky DNA adducts as well (12). The nucleotide excision repair pathway removes AFB1–dG adducts through the dual-incision mechanism to maintain genome integrity. If left unrepaired, AFB1–dG bulky adducts can cause mainly G to T transversions such as the mutation hotspot at codon 249 of the *TP53* gene found in HCC patients. The 3D genome organization functions in a variety of nuclear processes, including DNA replication, gene regulation, and DNA damage and repair. To better understand the molecular mechanism of AFB1 induced hepatocarcinogenesis, it is important to reveal the molecular details of AFB1-induced DNA damage formation and subsequent repair events within the 3D human genome organization. Previous studies focused on specific genes such as *TP53* gene by using conventional radioactive labeling-based methods including ligation-mediated PCR (LMPCR) (7,74). However, those studies that interrogated AFB1-induced DNA damage and repair in linear DNA sequences lack the information of 3D genome organization. It remains unknown how AFB1–dG adducts are repaired at genome-wide level and how 3D genome organization interplay with the AFB1–dG repair. Here, for the first time, we mapped the AFB1–dG repair across the whole genome with single-nucleotide resolution and revealed a heterogeneous AFB1–dG repair landscape across the four different genome organization levels: chromosome territories, A/B compartments, TADs, and chromatin loops.

Our adaptation of tXR-seq method has enabled us to create a genome-wide repair map of AFB1–dG adducts with single-nucleotide resolution in humans. This modified approach involves converting AFB1–N^7^–dG to ring-opened AFB1–FAPY–dG adducts and using an anti-AFB1 monoclonal antibody for additional purification during library construction. While previous studies have demonstrated high conversion efficiency for AFB1–N^7^–dG and high specificity and sensitivity of the anti-AFB1 monoclonal antibody (47), we acknowledge potential limitations. Some signal may be lost from excision products containing unconverted AFB1–N^7^–dG, and minimal non-specific signal could arise from antibody cross-reactivity. Despite these potential constraints, our method provides unprecedented insights into AFB1–dG repair across the 3D human genome structure.

As expected, we observed the preferential repair on TS of *TP53* gene, indicating that repair-resistant AFB1–dG adducts, like CPD lesions, are primarily eliminated through TCR subpathway. To validate the pivotal role of TCR in AFB1–dG repair, future studies using TCR-deficient cell models are needed. In our analysis, we revealed a distinct length distribution pattern of excision products released during AFB1–dG repair. The predominant excision product length is only 25 nt long, which is 1 nt shorter than previously identified excision products resulting from the repair of a variety of DNA bulky adducts caused by substances such as BPDE, EdU, cisplatin, and UV. Previous studies have shown that the TFIIH complex translocates along the ssDNA for lesion scanning (75), and the length of ssDNA affects the activity of endonucleases responsible for the dual incision (76,77). The sequence context and nucleosome position were found to regulate the dual incision site selection (78). Apart from the above factors, the TFIIH core complex may halt before the AFB1–dG at a position one nucleotide shorter than it does for other DNA lesions. However, further work is required to validate the precise dual incision reactions for repairing AFB1–dG adducts in humans.

Our in-depth analysis of AFB1–dG repair within the context of 3D genome organization provided several critical insights into the complex mechanisms underlying the dynamics of AFB1–dG repair. Integrating multiple datasets, including tXR-seq, DNA FISH, Hi-C and ChIA-PET, we revealed that AFB1–dG repair is heterogeneous across the chromosome territories, A/B compartments, TADs and chromatin loops. We observed the dependency of AFB1–dG repair on the spatial positioning of chromosomes within the nuclear architecture. Chromosomes located closer to the nuclear center, such as HSA-19, exhibited high repair compared to those situated in the nuclear periphery, like HSA-18. This finding implies a spatial regulation of repair activity within the cell nucleus. Furthermore, we identified the effect of A/B compartments on AFB1–dG repair. The A compartments, characterized by active and euchromatin regions, displayed higher AFB1–dG repair levels relative to the B compartments, which are associated with inactive and heterochromatin regions. In our previous work, we demonstrated that CPD and (6–4)PP repair super-hotspots are enriched in frequently interacting regions (FIREs) and super-enhancers (79). Here, we identified the enrichment of AFB1–dG repair in the vicinity of TAD boundaries and chromatin loop anchors. Interestingly, we found the corner score of TADs and loop strength are positively associated with repair levels, while there was a negative correlation with the size of these genome structures. These results suggest that genomic regions with robust transcriptional activity are favored for repair through the TCR subpathway. Meanwhile, epigenetic regulation, particularly histone modifications that maintain a euchromatin state, might contribute to the high global repair on NTS within regions exhibiting high TCR. The nucleosome-depleted regions flanking the CTCF binding sites might be more susceptible to DNA damaging agents, potentially resulting in higher damage formation levels. This increased damage could also contribute to the observed high repair activity in these regions. This complex interplay between DNA damage formation, repair, transcription, and chromatin structure underscores the intricate relationship between 3D genome organization and DNA repair processes.

Our observation of elevated AFB1–dG repair activity at TAD boundaries is consistent with the previous study on the relationship between nucleotide excision repair and higher-order chromatin structure in yeast (80). In that study, it was found that the origins of global genomic repair occur at the boundaries of chromosomal domains. These boundary regions might be more accessible to repair factors due to their unique chromatin environment, facilitating efficient damage detection and repair initiation. Although that study focuses on global genomic repair in yeast, the findings suggest that the impact of higher-order chromatin structure on repair activity may be a conserved feature across different species and nucleotide excision repair subpathways. Our results show that (6–4)PP repair levels do peak around TAD boundaries and loop anchors. However, these peaks are not as pronounced as those observed for CPD repair. We extend this concept to TCR of AFB1–dG adducts in human cells, highlighting the importance of considering the 3D genome organization when investigating DNA damage and repair processes.

Although TADs are highly conserved fundamental units of 3D genome organization, their structures are dynamic in cycling mammalian cells (81). When exposed to DNA-damaging agents, the DNA damage response and subsequent repair processes can potentially impact the 3D genome organization. It has been documented that TAD boundaries are strengthened, which is attributed to increased recruitment of the architectural protein CTCF after ionizing radiation (34). Previous studies have demonstrated that CTCF recruitment to double-strand break sites promotes homologous recombination repair and cohesion-mediated loop extrusion on either side of the break contributes to the formation of γH2AX and DNA damage response foci (82–84). It is plausible that AFB1 exposure may induce similar TAD boundary strengthening to facilitate efficient nucleotide excision repair.

Recent studies have shown that chromosomes located near the nuclear periphery are more susceptible to damage from UV radiation (28,57,58). These studies have indicated that both CPD and (6–4)PP are enriched in heterochromatin regions, suggesting that peripheral genome organization serves as a protective shield for nuclear center regions against UV-induced damage. Beyond the alteration of 3D genome organization in response to DNA damage, the distribution of DNA damage within various layers of 3D genome organization may also affect the repair process. UV damage repair data generated through our methods have been used to investigate the distribution of melanoma mutations and mutational landscape of the human genome (72,85–87). Our AFB1–dG repair data generated in this study can potentially improve our understanding of AFB1-induced liver mutagenesis and carcinogenesis. Our genome-wide repair map reveals preferential repair of AFB1–dG adducts in transcriptionally active regions. This pattern may explain regions with less efficient repair could be more prone to accumulating mutations. We have uncovered a complex interplay between 3D genome organization and AFB1–dG repair. This relationship helps identify genomic regions that are more susceptible to mutagenesis, potentially highlighting areas of the genome at higher risk for cancer-driving mutations.

To comprehensively dissect the interplay between 3D genome organization and the repair of AFB1–dG adducts, further investigations into the alterations of 3D genome organization in response to AFB1 exposure are needed. Additionally, profiling the distribution of AFB1–dG adducts formation and repair at multiple time points post-treatment within the 3D genome structure is required. These future studies will enhance our understanding of the dynamic relationship between DNA damage, repair, and 3D genome architecture, offering valuable insights into the mechanisms underlying AFB1-induced mutagenesis and carcinogenesis.

## Supplementary Material

gkae755_Supplemental_File

## Data Availability

The raw AFB1 tXR-seq data in this study were deposited in GEO (accession no. GSE243425). Scripts used in this article are available at https://zenodo.org/doi/10.5281/zenodo.12690500.
