# Supplementary material for: Nucleotide excision repair of aflatoxin-induced DNA damage within the 3D human genome organization
Source: Nucleic Acids Res. 2024 Sep 11;52(19):11704–19. doi: 10.1093/nar/gkae755 (PMC11514448; doi:10.1093/nar/gkae755)
Supplement: gkae755_Supplemental_File [file gkae755_supplemental_file.pdf]

Supplementary Materials for

# Nucleotide Excision Repair of Aflatoxin-Induced DNA Damage within the 3D Human Genome Organization

Yiran Wu et al.

Supplementary Figures 1-7

Corresponding author: Aziz Sancar, aziz_sancar@med.unc.edu; Wentao Li, wentao.li@uga.edu

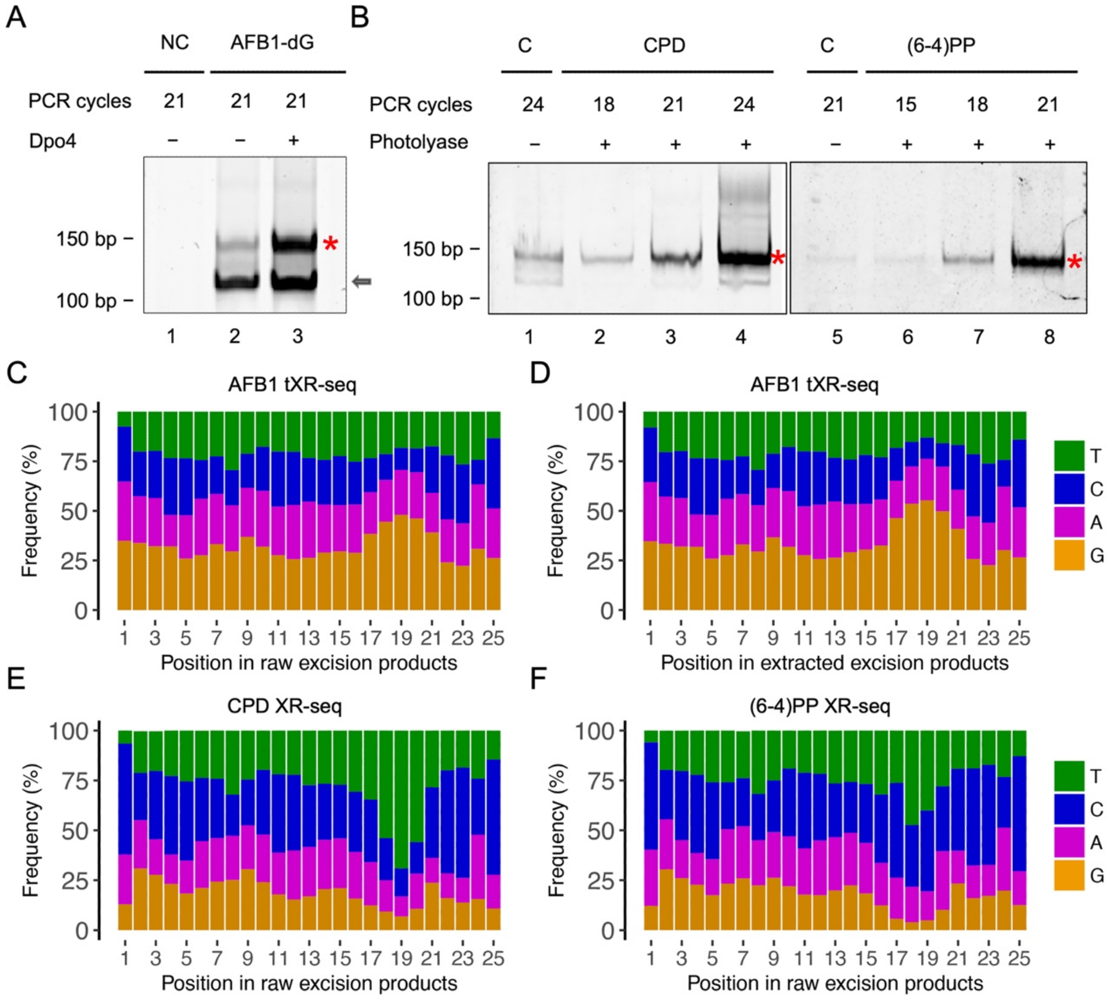


**Fig. S1.** Analysis of the dsDNA libraries and nucleotide frequencies of the excision products in AFB1-dG tXR-seq and CPD/(6-4)PP XR-seq. (A) Analysis of the dsDNA library for AFB1-dG tXR-seq through 10% native polyacrylamide gel electrophoresis, with the red asterisk indicating PCR products with inserts and the black arrow indicating adaptor-only PCR products. Dpo4 refers to sulfolobus DNA polymerase IV, a Y-family DNA polymerase known for its ability to bypass various DNA lesions, including AFB1-FAPY-dG bulky adduct. NC, non-template control. (B) Analysis of the dsDNA libraries for CPD/(6-4)PP XR-seq through 10% native polyacrylamide gel electrophoresis. C stands for control group in which photolyase is not present. For AFB1-dG tXR-seq, the single-nucleotide frequencies for the 25-mers in the raw excision products (C) and in the extracted excision products (D) after alignment to the human reference genome are shown. Single-nucleotide frequencies for the 25-mers of the raw reads obtained by CPD XR-seq (E) and (6-4)PP XR-seq (F) are also displayed. Frequency means the single nucleotide frequency at each position of the 25-mers.

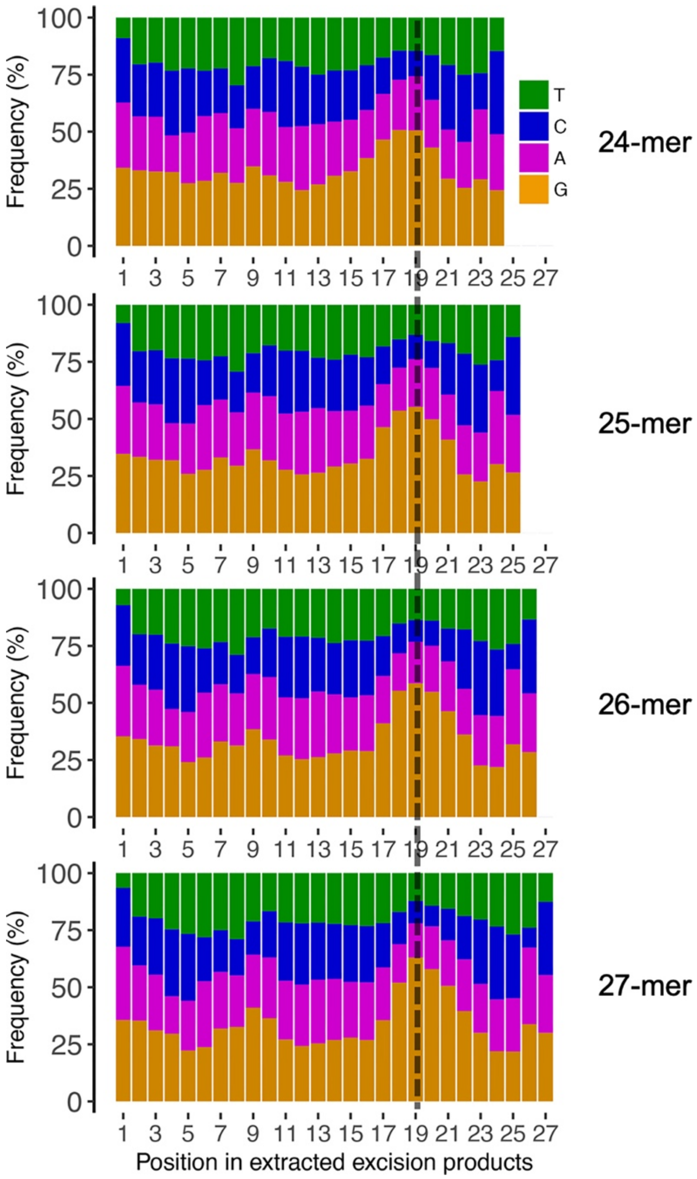


**Fig. S2.** Single-nucleotide frequencies at each position of 24-27-mers obtained through AFB1-dG tXR-seq extracted reads. Notably, the enrichment of Gs at position 19 is highlighted by a black dashed line.

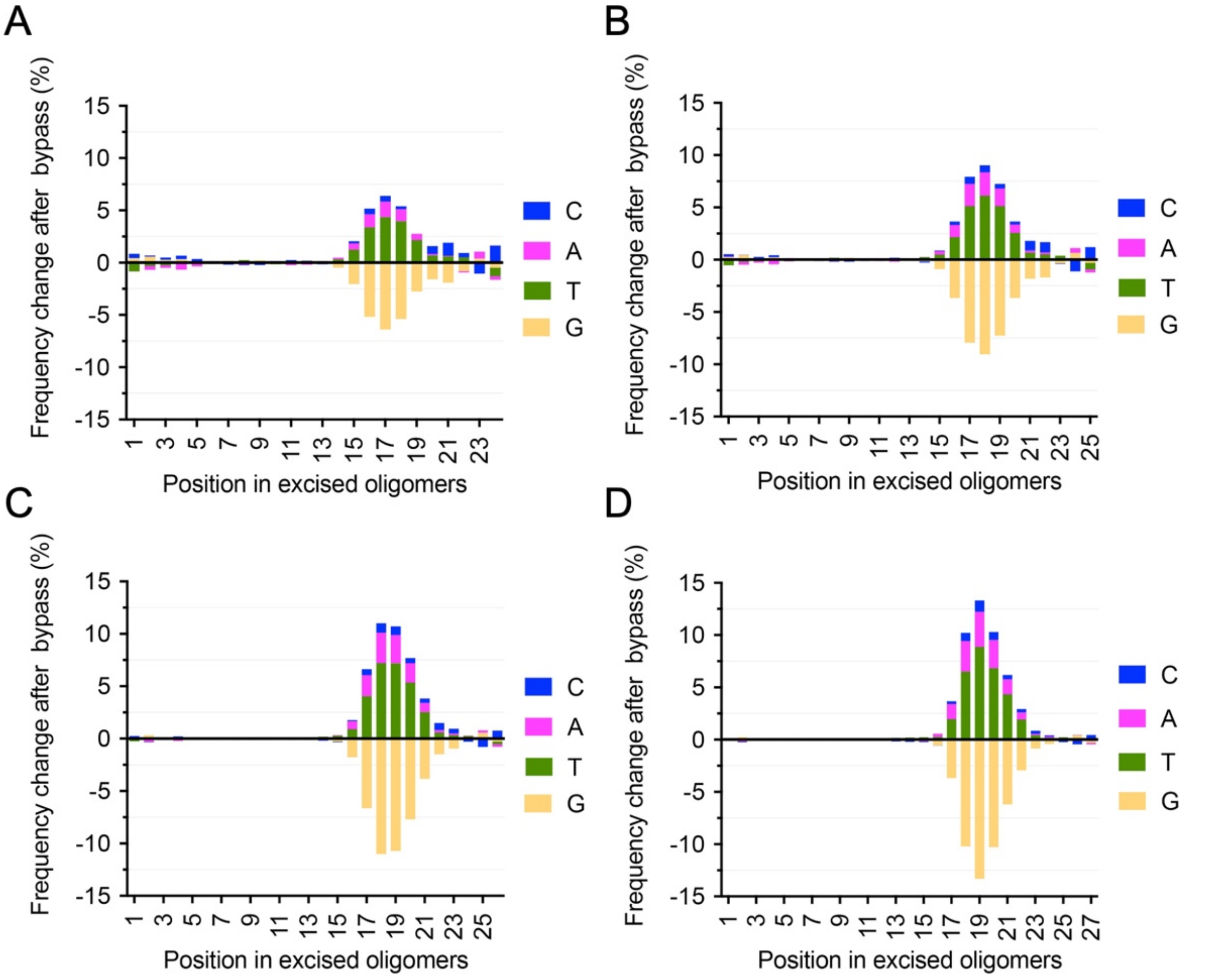


**Fig. S3.** Dpo4 primarily induces G to T transversions. The analysis focuses on single nucleotide frequency changes following bypass in excision products of different lengths, including 24-mers (A), 25-mers (B), 26-mers (C), and 27-mers (D). After bypass read sequences are extracted from the human reference genome after alignment and compared their nucleotide frequencies with those of the raw reads. Frequency means the single nucleotide frequency at each position of the excision products with certain lengths.

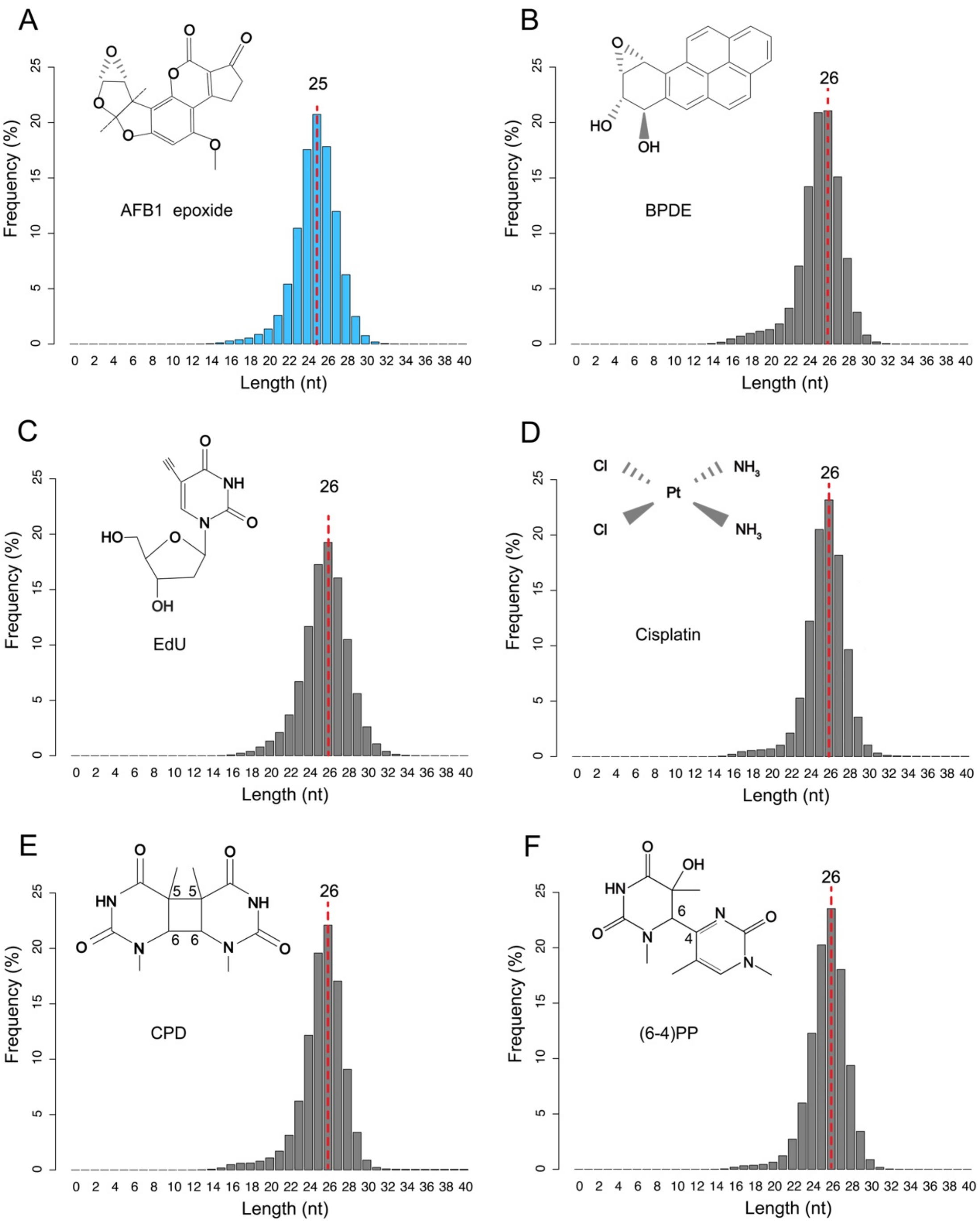


**Fig. S4.** Length distributions of excision products obtained through tXR-seq for AFB1-dG (A) and BPDE (B), and XR-seq for EdU (C), cisplatin (D), CPD (E), and (6-4)PP (F). The frequency refers to percentage of excision products with a specific length relative to the total number of reads. Excision products with the highest frequency are indicated by a red dashed line.

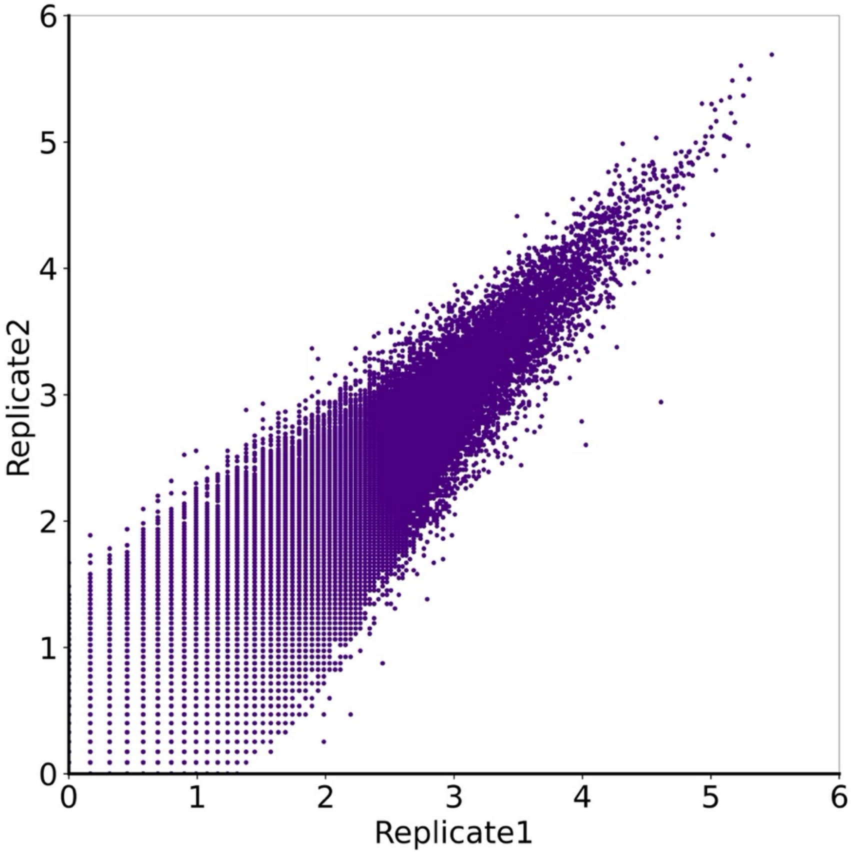


**Fig. S5.** Scatter plot showing correlation of excision repair signals between the two biological replicates (Replicate1 and Replicate2) for AFB1-dG tXR-seq. The values of X- and Y-axes are the $log_2$(normalized read counts). The value of Pearson *r* is 0.91.

.

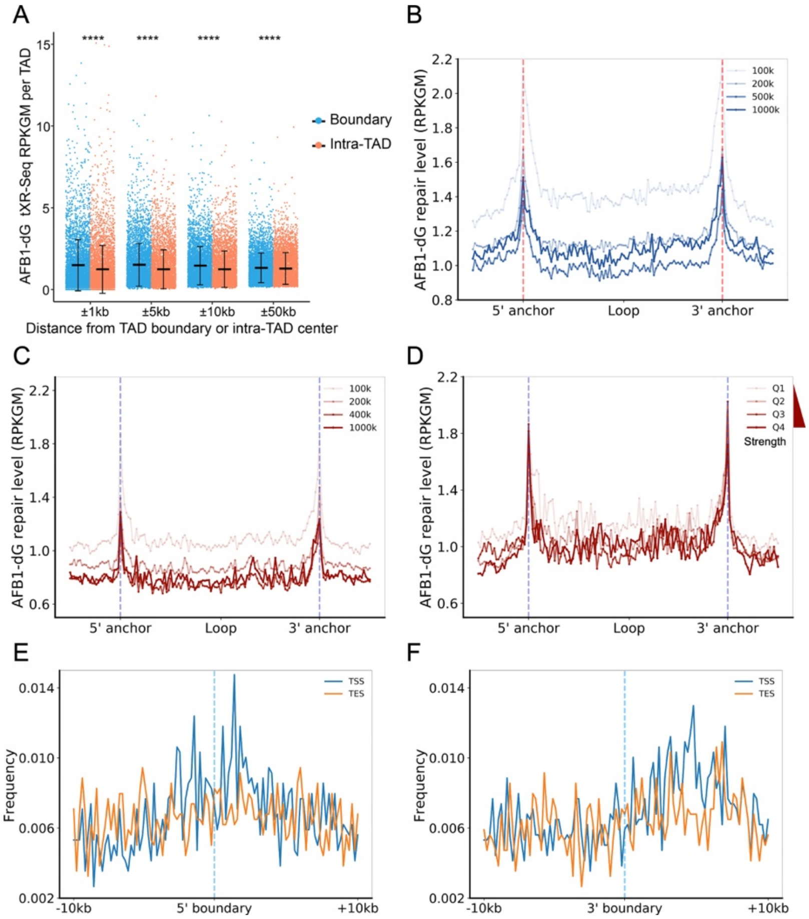


**Fig. S6.** Enrichment of AFB1-dG repair and transcription activity around both TAD boundaries and chromatin loop anchors. (A) AFB1-dG repair levels within extended regions (1kb, 5kb, 10kb, and 50kb) both upstream and downstream of TAD boundaries. Mean values with standard deviation (SD) are indicated with a black error bar. Statistical comparisons between mean values from both groups were conducted, and p-values were calculated using the Wilcoxon rank test. The p-values for ±1kb, ±5kb, and ±10kb are all less than 2e-16, and the p-value for ± 50kb is 5.6e-6. (B) AFB1-dG repair levels across chromatin loops with different lengths from HiC data from HepG2 cells. (C) AFB1-dG repair levels across chromatin loops with different lengths obtained from CTCF ChIA-PET data. (D) AFB1-dG repair levels across 2737 chromatin loops (length < 100 kb) obtained from CTCF ChIA-PET data. The chromatin loops were categorized into quartiles based on loop strength, ranging from Q1 (lowest strength) to Q4 (highest strength). (E) Frequency distributions of TSS and TES surrounding the 5' boundary regions of 4311 nonoverlapping TADs are displayed. (F) Frequency distributions of TSS and TES surrounding the 3' boundary regions of 4311 nonoverlapping TADs are shown.

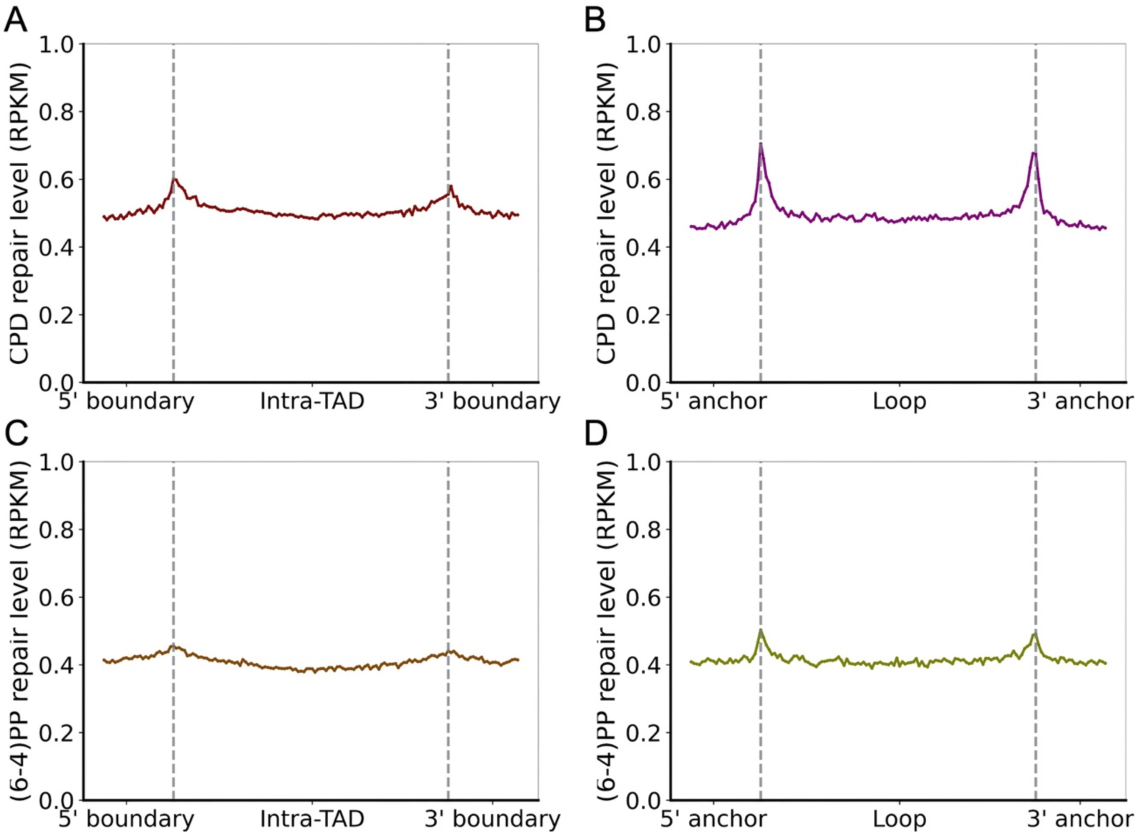


**Fig. S7.** CPD and (6-4)PP repair levels across nonoverlapping TADs and chromatin loops. (A) Average CPD repair level over 4311 nonoverlapping TADs. (B) Average CPD repair level over 2989 nonoverlapping chromatin loops that are less than 200 k. (C) Average (6-4)PP repair level over 4311 nonoverlapping TADs. (D) Average (6-4)PP repair level over 2989 nonoverlapping chromatin loops that are less than 200 kb. TAD boundaries and loop anchors are indicated by grey dashed lines.
